# The federal government and Canada's COVID-19 responses: from ‘we're ready, we're prepared’ to ‘fires are burning’

**DOI:** 10.1017/S1744133121000220

**Published:** 2021-06-22

**Authors:** Sara Allin, Tiffany Fitzpatrick, Gregory P. Marchildon, Amélie Quesnel-Vallée

**Affiliations:** 1 Institute of Health Policy, Management and Evaluation, University of Toronto, Toronto, ON, Canada; 2 North American Observatory on Health Systems and Policies (NAO), University of Toronto, Toronto, ON, Canada; 3 Yale School of Public Health, Yale University, New Haven, CT, USA; 4 Departments of Epidemiology, Biostatistics and Occupational Health and Sociology, McGill University, Montreal, QC, Canada; 5 McGill Observatory on Health and Social Services Reforms, Montreal, QC, Canada

**Keywords:** Canada, COVID-19, federalism, public health

## Abstract

Canada's experience with the coronavirus disease-2019 (COVID-19) pandemic has been characterized by considerable regional variation, as would be expected in a highly decentralized federation. Yet, the country has been beset by challenges, similar to many of those documented in the severe acute respiratory syndrome outbreak of 2003. Despite a high degree of pandemic preparedness, the relative success with flattening the curve during the first wave of the pandemic was not matched in much of Canada during the second wave. This paper critically reviews Canada's response to the COVID-19 pandemic with a focus on the role of the federal government in this public health emergency, considering areas within its jurisdiction (international borders), areas where an increased federal role may be warranted (long-term care), as well as its technical role in terms of generating evidence and supporting public health surveillance, and its convening role to support collaboration across the country. This accounting of the first 12 months of the pandemic highlights opportunities for a strengthened federal role in the short term, and some important lessons to be applied in preparing for future pandemics.

## 1. Introduction

Through its federal, provincial, territorial (FPT) and municipal governments, Canada had the plans, experience and infrastructure to effectively manage and respond to a pandemic. However, Canada's performance during the coronavirus disease-2019 (COVID-19) pandemic, especially after the first wave, reveals several shortcomings and areas for strengthened responses. COVID-19 fatalities disproportionately affected vulnerable populations in Canada – in particular, residents of long-term care (LTC) facilities – and, throughout Spring 2021, hospitals in many regions were on the verge of being overwhelmed as a result of the second and third pandemic waves. In comparison with its southern neighbors in the Americas, namely the United States and Mexico, the Canadian experience appears to have been a relative success (Béland *et al*., 2020b). However, comparisons with exemplars during the COVID-19 pandemic, such as Australia, New Zealand and South Korea, highlight shortcomings in Canada's pandemic preparedness and responses.

The significant regional variation in the pandemic experiences across Canada, along with widespread limitations with all five components of the ‘find, test, trace, isolate and support’ system, point to systemic challenges beyond those inherent to a highly decentralized federation (Marchildon, 2018). Experiences with the 2003 severe acute respiratory syndrome (SARS) epidemic and 2009 H1N1 pandemic created opportunities for Canada to refine and test its pandemic response plans. Significant efforts were deployed by FPT governments to learn from these experiences and recommendations following various reports and analyses (Webster, 2020), leading to concrete achievements such as the establishment of the Public Health Agency of Canada (PHAC) in 2006, a dedicated federal public health agency strengthening the role of the federal government in public health (Health Canada, 2003). The 2018 Canadian Pandemic Influenza Preparedness Plan, based on the principles of *Collaboration, Evidence-Informed Decision Making, Proportionality,* and *Flexibility*, was the culmination of many of the lessons learned from these earlier experiences and provided the playbook for the COVID-19 pandemic response (PHAC, 2018); however, reports unveiled that this new plan had not been fully tested before the COVID-19 pandemic (Ontario Auditor General, 2020; Robertson, 2020).

This paper critically reviews Canada's experiences with the COVID-19 pandemic, as of the end of March 2021, with a focus on the role of the federal government in supporting the public health actions of ‘find, test, trace, isolate and support’ (Rajan *et al*., 2020). As a primarily descriptive historical accounting of the first year of the COVID-19 pandemic and, given that at the time of writing the country was experiencing a major third wave, we are unable to provide a definitive assessment of the optimal role for the federal government in the pandemic. However, our broad aim is to shed light on potential areas where a strengthened federal role may be warranted in a public health emergency. The paper is structured as follows: first, we provide a brief overview of the first year of the COVID-19 pandemic experience in Canada; second, we pay attention to the key areas where the federal government plays – or could play, in the case of LTC – a major role in the public health response and finally, we consider emerging lessons learned and considerations for the role of the federal government in managing public health emergencies in the context of a decentralized federation.

## 2. COVID-19 in Canada: public health structure and epidemiologic trends

As a highly decentralized federation, public health measures in Canada are the shared responsibility of FPT governments. Although the federal government is responsible for a few other designated public health measures and public health surveillance, PT governments are primarily responsible for most pandemic emergency responses and the administration of their tax-funded universal health insurance programs. PT governments have further delegated some public health responsibilities to dedicated provincial and regional public health agencies (Supplementary materials, Table 1). Most PT – and many local – governments also formed dedicated COVID-19 response teams, responsible for advising and assisting political leaders with decisions regarding public health and economic measures. Overall, the emergency management framework in Canada positions the federal government largely in a coordinating and convening function, with provinces taking the lead and only requesting support from the federal government when needed (Fierlbeck, 2010; Migone, 2020). However, there are areas where the federal government is primarily responsible, such as border control, discussed at further length below.

The first confirmed case of COVID-19 in Canada – a traveler from Wuhan, China – was identified in Toronto, Ontario on 25 January 2020 (CCODWG, 2021). Throughout February 2020, Canada's COVID-19 case count grew slowly, consisting mostly of travelers from international hotspots. It did not take long, however, for case counts to rise more rapidly, as community transmission became increasingly widespread during the spring. Cumulatively, the absolute number of COVID-19 cases and deaths have been concentrated in Ontario and Quebec – the two most populous provinces, located in central Canada (CCODWG, 2021).^1^^,^^2^

Early in the pandemic, the Pacific coast province of British Columbia (BC) was widely considered a local success – it experienced outbreaks early, but swiftly and seemingly effectively implemented containment measures (Porter, 2020; Young, 2020). However, BC has since joined other PTs in experiencing subsequent waves substantially larger (in terms of cases) than the first. Although many PTs went weeks, some months, without new cases during the summer months,^3^ by December 2020, most were already experiencing second wave magnitudes greater than the first. Throughout the second wave, record numbers have been set almost daily and new provinces have emerged as COVID-19 hotspots.^4^ Moreover, mounting evidence has shown that the pandemic has disproportionately affected women and racialized persons across Canada – both directly, in terms of COVID-19-related health burden, and indirectly, as a result of the economic consequences of public health measures (Béland *et al*., 2020b; PHAC, 2020i).^5^

## 3. Areas requiring federal leadership in public health

The federal government in Canada is uniquely placed to fulfill some key public health functions during a public health emergency. Overall, the federal government has, to varying degrees, played a role in (1) mitigating viral transmission through the use of international border controls and travel bans, albeit with major implementation challenges; (2) supporting PT test, trace, isolate and support efforts with guidelines and emergency funding, despite the absence of any national data system and, (3) communicating with the public to increase support for public health measures and unite the country toward a shared understanding of the crisis and the personal measures required to minimize contagion, although these lacked coordination with, and in some cases were overshadowed by, PT-level public health communication.

### 3.1 Border control and travel bans

Border control, including closures and mandatory quarantine, is unambiguously primarily a federal responsibility. Although the first official communication from the country's most senior public health officer, Chief Public Health Officer (CPHO) Dr Tam, was on 20 January 2020, the increased risk to Canadians was not recognized – or at least not publicly acted upon – until March 2020, when a number of travel-related measures and recommendations were introduced (PHAC, 2020a). The country's travel bans have been considered to be a major contributor to Canada's ability to contain the pandemic during the first wave.^6^ On 16 March 2020, the federal government rerouted most^7^ commercial international arrivals to four airports across Canada (Vancouver, Calgary, Toronto and Montreal) (Canada, 2020a). Two days later, all international borders were closed to the majority of foreign nationals, prohibiting all ‘non-essential travel’, making Canada one of the first countries to implement a total border closure (Hoffmann and Fafard, 2020; PHAC, 2020d). This was followed by restrictions to non-essential travel across the USA–Canada land border on 21 March 2020 (still in effect as of April 2021) and a mandatory 14-day unsupervised quarantine for all individuals entering Canada, enacted under the *Quarantine Act*, on 25 March 2020 (PHAC, 2020b).

However, this border closure may have come too late for some provinces, which exhibit substantially different travel patterns. Many Canadians travel abroad in March – school children across the country have a week-long break – and common destinations include the United States and the Caribbean (BCCDC, 2020b); BC has a large Asian community, thus Asia-Pacific locations are also common destinations, while European destinations are common for French-speaking Quebec (BCCDC, 2020b; Murall *et al*., 2020). Quebec's spring break was the earliest among the most populous provinces, spanning the weeks of 29 February to 9 March 2020, and genomic analyses show the majority of cases identified during the first wave in Quebec were linked to Europe, the Caribbean, and the United States, but rarely from Asia – despite being the focus of Canada's early travel bans (Murall *et al*., 2020).

The Canadian approach to border control has also been criticized for its multiple exemptions, and perhaps more importantly, the weak enforcement of the mandatory quarantine for incoming travelers, including (until February 2021) the absence of any testing requirements (Hill and Russell, 2021; Tunney, 2021). For example, as of 26 January 2021, only 48,682 incoming travelers, approximately 2.4% of all international travelers required to quarantine under Canada's *Quarantine Act*, had been followed up by PHAC or a law enforcement agency to monitor compliance (Tunney, 2021); only 141 fines were issued for non-compliance (ibid). On 29 January 2021, Transport Canada announced it had contracted four security companies to complete compliance checks in 35 cities across the country (Canada, 2021c). Notably, approximately 74% of international travelers are essential workers exempt from the *Quarantine Act* (Hill and Russell, 2021). Nearly one year after the first confirmed case of COVID-19 in Canada, the border closure was strengthened with a mandatory negative test being required for all inbound travelers (72 h prior to departure/arrival for air and land border crossings), limiting all international flights to four designated airports (with very limited exceptions), the cancellation of airline service to popular sun destinations (in collaboration with four of the country's largest airlines), mandatory testing upon arrival (and near the end of quarantine) and a mandatory supervised three-day quarantine in a Government of Canada-approved hotel for inbound air travelers, at the traveler's expense, followed by the completion of the mandatory isolation period in a designated government-run isolation facility for all travelers with a positive test result (PHAC, 2021a). Yet, these have faced ongoing criticism for the weak adherence and enforcement; for example, a federal report from early 2021 found the national public health agency was unable to assess adherence to the quarantine orders that had been in place since March 2020 for two-thirds of incoming travelers (Auditor General of Canada, 2021; Canada, 2021c). As of 19 April 2021, over 179,000 travelers had reportedly received an in-person visit to verify compliance – much greater than the number reported earlier in the pandemic – with law enforcement following up with over 80,000 individuals and issuing 801 tickets for failure to comply, including 404 for failure to stay in a mandatory quarantine hotel upon arrival (Neustaeter, 2021).

**Figure 1.**
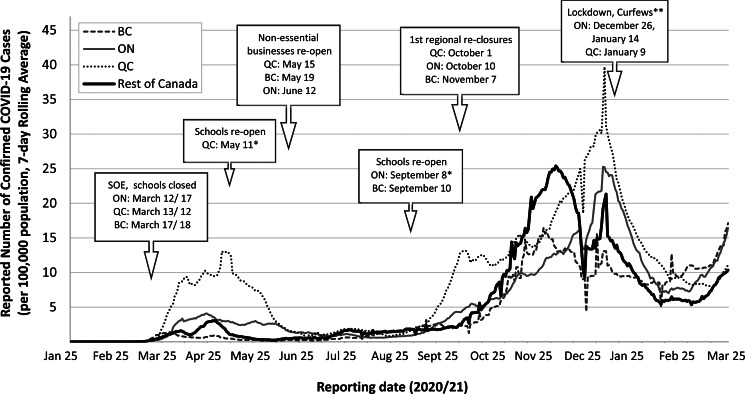
Reported daily number of confirmed COVID-19 cases, per 100,000 population (7-day rolling average),^1^ reported in British Columbia (BC), Ontario (ON), Quebec (QC) and the rest of Canada, as well as the timing of relevant public health measures. *Opening dates varied by region. In QC, schools in the Greater Montreal region, representing approximately 50% of QC's population, were not permitted to re-open for in-person instruction until 27 August 2020. In ON, schools in the Ottawa region, re-opened on 3 September 2020. **On 26 December 2020, a province-wide lockdown consisting of bans on gatherings, closure of non-essential businesses, etc. came into effect in ON. On 9 January 2021, QC introduced a curfew, restricting non-essential travel outside the home between the hours of 8pm and 5am. On 14 January 2021, a stay-at-home order came into effect in ON, restricting non-essential trips outside of the home, and a new state of emergency was declared. SOE: Declaration of State of Emergency or Public Health Emergency, resulting in the closure of non-essential businesses and restrictions on the size of gatherings. ^1^Data source for reporting dates 25 January 2020–31 March 2021: CCODWG (2021). COVID-19 Canada Open Data Working Group (CCODWG) Dataset. https://opencovid.ca/work/dataset/. Population sizes (2019): Canada – 37,589,262; QC – 8,484,965; ON – 14,566,547 and BC – 5,071,336. Statistics Canada (2021). Annual Demographic Estimates: Canada, Provinces and Territories, 2019. https://www150.statcan.gc.ca/n1/pub/91-215-x/91-215-x2019001-eng.pdf.

Complementing these federal measures were varied efforts by PT and local governments to restrict domestic travel restrictions. The most successful has been the ‘Atlantic bubble’, which allowed travel between the four Atlantic provinces but did not permit residents of other PTs entry, except for extraordinary circumstances (Flood and Thomas, 2020); anyone permitted to enter from outside the bubble was required to undergo a mandatory 14-day quarantine, and provide adequate details for their quarantine plan prior to entry (PEI, 2020). Outside the Atlantic region, domestic travel restrictions were rarely used in the first year of the pandemic, although the border between Ontario and Quebec was closed periodically to non-essential travel, and there were some efforts to reduce within-province travel in BC and Ontario in early 2021 in response to the third wave. Figure 1 shows the timing of the major public health measures introduced in BC, Ontario and Quebec, relative to the daily reported number of cases per capita (public health measures are further described in Supplementary materials, Table 2).

### 3.2 Support PT test, trace, isolate and support systems with guidelines and emergency funding

**Figure 2.**
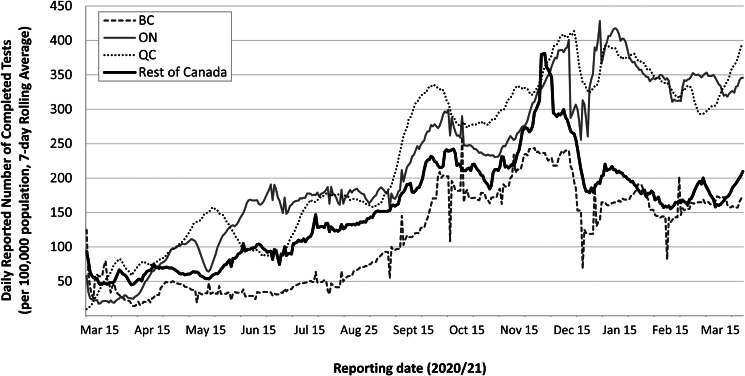
Reported daily number of completed SARS-CoV-2 diagnostic tests, per 100,000 population (7-day rolling average),^1^ reported in British Columbia (BC), Ontario (ON), Quebec (QC) and the rest of Canada. ^1^Data source for reporting dates 15 March 2020–31 March 2021: CCODWG (2021). COVID-19 Canada Open Data Working Group (CCODWG) Dataset. https://opencovid.ca/work/dataset/. Data for Quebec retrieved from INSPQ (2021). COVID-19 data in Quebec. https://www.inspq.qc.ca/covid-19/donnees. Population sizes (2019): Canada – 37,589,262; QC – 8,484,965; ON – 14,566,547 and BC – 5,071,336. Statistics Canada (2021). Annual Demographic Estimates: Canada, Provinces and Territories, 2019. https://www150.statcan.gc.ca/n1/pub/91-215-x/91-215-x2019001-eng.pdf.

Throughout the pandemic, the federal government leveraged both its technical capacity and convening function to develop technical guidance and contribute to data and information sharing of best practices to support PT governments with the core public health functions of infectious disease control. For example, the federal government released its first guidance document for COVID-19 testing indications on 16 April 2020 (PHAC, 2020h) – since updated numerous times – and has also provided guidance on contact tracing (PHAC, 2020c). Although federalism provides PT governments flexibility to respond to the unique needs of each region, uncoordinated efforts may have prevented the country as a whole from effectively using its resources (Desson *et al*., 2020; Flood and Thomas, 2020). For instance, the first report from the federal COVID-19 Testing and Screening Expert Advisory Panel, published on 15 January 2021, noted the need to strengthen PT collaboration to more effectively manage the pandemic (Health Canada, 2021). Such collaboration could help overcome the wide variations in testing capacity across the country given only an estimated ‘75 per cent of the national capacity is used on average each day’ (Health Canada, 2021: p. 9); while some PTs had a growing backlog in unprocessed tests, others regularly did not use their available laboratory capacity. Similarly, a review of Canada's pandemic response by the Canadian Public Health Association (CPHA) recommended a pan-Canadian framework for testing protocols, testing capacity and laboratory surge capacity could strengthen the existing national pandemic response plan (CPHA, 2021). Initially, a limited supply of test kits, and the restriction that all tests be performed or certified by the National Microbiology Laboratory in Winnipeg, resulted in a scarcity of tests and slow turn-around times across the country. Although PTs eventually ramped up their testing capacities, the process was slow and remained inadequate relative to demand (see Figure 2). For example, although Ontario drastically scaled up its testing capacity, it only achieved its target of 50,000 tests per day on 26 November 2020 (CCODWG, 2021). Similarly, Quebec set a goal of processing 20–30,000 tests per day in mid-April but did not achieve this until 9 September 2020 (INSPQ, 2021; MacLellan, 2020). Test results had been typically available within 48 h in BC and Quebec, as of January 2021 (Quebec, 2020; BCCDC, 2020a); while, in Ontario, test turn-around times regularly exceeded four days between March and August 2020 (Ontario Auditor General, 2020). Arguably, a strengthened federal role in collaboration and information sharing could have addressed some of these shortcomings, and helped bolster capacity to meet the demands and ensure adequate testing capacity across the country. Moreover, the highly variable approach to the use of rapid diagnostic tests,^8^ first approved by Health Canada in late March 2020, signals further need for federal support in terms of guidelines and protocols (PHAC, 2020k).

Moreover, despite limited data to assess the adequacy of contact tracing and testing across Canada due to a lack of a pan-Canadian electronic public health surveillance system (Bhatia *et al*., 2020),^9^ the federal government provided minimal supports to PTs to bolster their overstretched public health workforce. The federal exposure notification mobile ‘app’ was initially rolled out in Ontario on 31 July 2020, and was taken up in all but four PTs as of April 2021. However, it had limited uptake nationally, with just over 6.3 million downloads as of February 2021, representing about 17% of the Canadian population, and estimates suggest fewer than 5% of positive cases entered their positive test result into the app (Canada, 2021a, 2021b). Moreover, limited capacity for manual contact tracing in the PTs meant contacts were often not notified of potential exposure in a timely manner, one activity these exposure notification apps were intended to support, nor were tracers able to identify the source of infection for the majority of cases. For example, as of 31 March 2021, 27% of laboratory-confirmed COVID-19 cases in Ontario had no confirmed epidemiological link (Public Health Ontario, 2021) and, in Fall 2020, approximately 25–35% of known cases did not respond to contact tracing efforts in Quebec (Lauzon, 2020); no such statistics currently exist at a national level.

Furthermore, the federal government played some role, mainly in terms of emergency funding, to ensure there are adequate supports for isolation for those in need, in spite of provincially mandated isolation requirements for those confirmed to be infected with severe acute respiratory syndrome coronavirus 2 (SARS-CoV-2). One region of Ontario – Peel, one of Canada's most ethnically diverse demographics and home to many high-risk workplaces (e.g. large manufacturing and retail distribution centers, Canada's largest international airport, etc.) – experienced the greatest proportion of Ontario's cases during the second and third waves, and test positivity among many neighborhoods regularly exceeded 20% (ICES, 2021). In response, Peel Region's three mayors issued a joint letter asking the federal government to finance new isolation centers; within hours, the federal government provided CA$6.5 million to support their request (PHAC, 2020j). Peel's was one of nine voluntary culturally diverse isolation centers in Ontario (as of February 2021), which had received CA$39 million in federal support as of 8 February 2021 (King, 2021); the majority of these sites opened throughout December 2020 and January 2021 (ibid). As of 8 February 2021, the federal government reportedly operated 11 quarantine hotels, the majority of which were located in Ontario, and two joint federal-provincial sites (Bresge, 2021). In March 2021, funding was announced for two additional sites in Ontario, including a dedicated isolation and recovery center for temporary foreign agri-farm workers (Ontario, 2020c; PHAC, 2021b). Through its CA$100 million Safe Voluntary Isolation Sites Program, the federal government filled gaps in PT funding to provide free voluntary isolation/quarantine rooms for travelers with inadequate quarantine plans, other residents needing a safe place to quarantine/isolate, and, in the case of Nunavut, travelers required to quarantine before permitted entry into the territory; notably, of this funding, CA$72.2 million was provided to municipalities in Ontario to operate 2140 beds, as reported on 30 March 2021 (Ontario, 2020c).

Although federal guidance and funding was provided to support infection prevention and control in shelters and other communal settings (PHAC, 2020f) to increase physical distancing and provide spaces for persons needing to isolate, the adequacy of these measures has varied across the country. For example, on 7 April 2020, the BC government announced it had secured 900 beds at 23 hotels, motels and other sites to support persons experiencing homelessness and other vulnerable persons requiring a safe place to quarantine/isolate (BC, 2020). The city of Montreal (Quebec) also implemented similar measures in Fall 2020 (although not on the same scale), converting two hotels and a former hospital wing to shelter spaces (Schwartz, 2020).

### 3.3 Coordinating pandemic communication

Given the scale and scope of the pandemic, the federal government is well placed to serve as a key spokesperson in public health communication. However, limited national and interregional coordination of public health communication was apparent throughout the pandemic, and the federal government fell short in leveraging its unique position to unite the public in supporting measures that help mitigate the pandemic.

Beginning 13 March 2020, Prime Minister Justin Trudeau delivered almost daily media updates to the public throughout the first wave of the pandemic (CPAC, 2021); by 29 June 2020, the Prime Minister had delivered 81 national addresses over 110 days (Aiello, 2020). Starting July 2020, these occurred with less frequency; however, throughout November and December 2020, these were again held on a near-daily basis as the country faced a more threatening second wave (PM Trudeau, 2021). The Prime Minister's update was regularly followed by a second press briefing from national public health authorities, including CPHO Dr Theresa Tam; these also occurred daily during the first wave and throughout November and December 2020. The Prime Minister and CPHO also consistently used social media, such as Twitter, to communicate their vision of tackling the pandemic by flattening the curve (e.g. #PlankTheCurve) and following public health advice (e.g. #StayAtHome).

Most PT governments also decided to provide daily updates, generally with some combination of the premier (i.e. the head of PT government) and public health and/or health authorities. Although early in the pandemic there was evidence of consistent messaging across the country, for instance by the FPT chief medical officers of health between January and March 2020 (Fafard *et al*., 2020), there was also some indication the advice given across FPT briefings was occasionally contradictory (Fierlbeck and Hardcastle, 2020). At best, this created confusion regarding the most up-to-date and geographically relevant messaging, further amplified by social media (ibid). At worst, inconsistencies propagated mis- and dis-information, as well as distrust, in public authorities (Fierlbeck and Hardcastle, 2020; Ontario Auditor General, 2020; PHAC, 2020i). For example, one study found a difference in take-up of federal (PHAC) guidance on mask use among residents of Quebec compared to the rest of Canada, which the authors attributed to the difference in PHAC and Quebec guidelines at the time, as well as more limited attention paid to federal compared to provincial health officials in Quebec (Sheluchin *et al*., 2020).

Moreover, the communication coming out of the federal government, whether by the Prime Minister or CPHO, appears to have fallen short in galvanizing public support for public health measures. However, some PT public health leaders appeared to have some success with effectively public communication in their jurisdictions. BC's top public health official, Dr Bonnie Henry, received both national and international accolades for her clear and empathetic communication style throughout the pandemic; a factor many suggested might have helped BC successfully manage the first wave of the pandemic (Porter, 2020). Similarly, Quebec's top public health official, Dr Horacio Arruda, generally received broad public support across the province; markedly, Dr Arruda sought the help of a communications coach in May and November 2020, to support his effective communication with the public (Authier, 2020). Although parts of the country, particularly Atlantic Canada, appeared to adopt the ambitious goal of eliminating the virus (or ‘COVID-zero’) early on in the pandemic (Austen, 2020), that the federal government did not leverage its voice in galvanizing public support for public health measures, nor in setting an ambitious vision to fight the pandemic, suggests a missed opportunity.

## 4. Long-term care in the provinces and territories: is pan-Canadian oversight needed?

Although the federal government financed the majority of COVID-19-related spending for public health and health systems measures – as well as broad economic relief measures – in response to the pandemic that fall under provincial jurisdiction, the LTC sector provides a notable exception where the federal government has no jurisdiction but where further leadership may be warranted. During the first wave of the pandemic, Canada experienced the highest proportion of COVID-19 deaths in LTC than any other Organization for Economic Co-operation and Development (OECD) country – 81%, almost twice that of the OECD average, 42% (Science Table, 2020). As of 22 November 2020, 75% of COVID-19 deaths in Canada had occurred among LTC residents, with 56% occurring in Quebec (NIA, 2020).^10^

**Figure 3.**
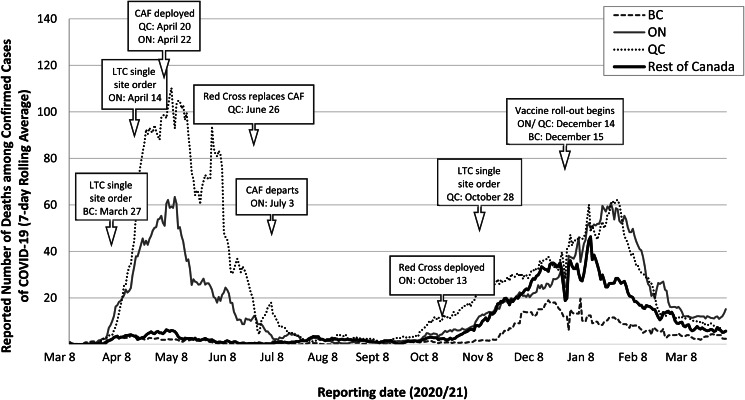
Reported daily number of deaths among confirmed cases of COVID-19 (7-day rolling average)^1^ reported in British Columbia (BC), Ontario (ON), Quebec (QC) and the rest of Canada, as well as the timing of relevant measures specific to LTC.^2^ ^1^Data source for reporting dates 8 March 2020–31 March 2021: CCODWG (2021). COVID-19 Canada Open Data Working Group (CCODWG) Dataset. https://opencovid.ca/work/dataset/. ^2^Although daily COVID-19 case and death data specific to LTC facilities are available for ON and QC, these are not available for BC (or for Canada as a whole). CAF, Canadian Armed Forces.

In both Ontario and Quebec, the federal government played a key role in managing outbreaks in the most afflicted LTC facilities by sending the Canadian Armed Forces (CAF) to support staff with infection control, cleaning and food preparation in April 2020. In May 2020, the CAF released a report detailing the deplorable and gruesome conditions witnessed in the affected facilities, including serious infection prevention, safety, care and staffing concerns (CMFM, 2020). Subsequently, Ontario launched an independent investigation into the conditions of their LTC facilities and the disproportionate impact of the pandemic (Ontario, 2020d). The federal government also issued guidance on infection control and prevention in LTC in April 2020 (most recently updated in February 2021) (PHAC, 2020e), yet these were insufficient to address the surge in cases and deaths in LTC, in particular in Ontario and Quebec. Moreover, in spite of a similar financing model for LTC across the provinces, BC did not experience as high a death toll from COVID-19 in its facilities due to a number of factors, including: better coordination between LTC facilities with hospitals and public health authorities; relatively more generous funding of LTC; enhanced resident care and LTC standards; more single rooms and non-profit ownership and more comprehensive inspections (Liu *et al*., 2020; Tuohy, 2020). In Quebec, the crisis in LTC has been attributed to longstanding LTC budget constraints, a province-wide worker shortage exacerbated by poor working conditions, and an increasing role for private providers with limited oversight aside from compliance with basic safety rules (Béland *et al*., 2020b). Figure 3 provides a comparison of all COVID-19-related deaths occurring in BC, Ontario and Quebec; however, detailed data regarding deaths occurring in LTC is not publicly available for all PTs, including BC.

Consistent with the federal guidelines, throughout the pandemic all PTs implemented visitation restrictions, including cohorting^11^ of infected/symptomatic LTC residents, expanded testing, increased LTC staff wages (to various degrees) and implemented orders restricting the movement of staff between multiple facilities (Supplementary materials, Table 3). For example, in Quebec, a complete lockdown was enforced, which prohibited residents from leaving LTC facilities and from having visitors during the peak of the first pandemic wave (MSSS, 2020).

However, poor infection prevention and control and inadequate staffing were major problems in both Ontario and Quebec, as reported by CAF (CAF, 2020; CMFM, 2020). Moreover, overcrowding of LTC facilities was a contributing problem in both provinces. A January 2021 analysis suggested 19% of infections and 18% of deaths in Ontario's LTC facilities could have been prevented if all four-person rooms had been converted to double occupancy (Science Table, 2020); 31 and 30%, respectively, could have been potentially prevented by converting to single occupancy (ibid). Although cohorting of infected residents was recommended in all regions, limited efforts or recommendations were made to increase the proportion of single-bed rooms (Canada, 2020b); many LTC facilities across Canada have outdated infrastructure and use a ward-style accommodation design with few private rooms and many shared communal spaces (Rankin, 2020).

In response to growing calls for a strengthened federal role in LTC, both in terms of financing and in oversight of national standards, the federal government pledged a five-year CA$3 billion investment (i.e. over 2022–2027) to Health Canada, in its 2021 budget, to support PTs in ensuring standards for LTC are met and permanent reforms to LTC occur (Canada, 2021d). Among those supporting the need for national standards were: the medical and nursing profession (CMA, 2020); a special task force of the federal government's Chief Science Advisor (Canada, 2020b); and other think tanks and advocacy groups, such as the Canadian Centre for Policy Alternatives (Armstrong and Cohen, 2020) and CARP, formerly the Canadian Association of Retired Persons (CARP, 2020). However, the roll out of this initiative would undoubtedly be met with resistance by some PT leaders, particularly those who strongly view health care as exclusively PT jurisdiction (Rabson, 2020). For example, in December 2020, the Quebec National Assembly unanimously rejected a federal plan to impose national standards as a condition on the PTs' access to increased federal funding for LTC and other residential care (The Canadian Press, 2020).

## 5. Reflections on the first year of the COVID-19 pandemic in Canada

The experiences in Canada over the first year of the COVID-19 pandemic suggest the federal government served a vital role in the pandemic largely by leveraging: (1) its authority to directly contain spread through border closures and travel bans; (2) its technical capacity to develop national guidelines to support PT efforts and (3) its leadership role in communicating with the public. Also, in the case of the crisis in LTC, the federal government, through CAF, played a direct role in managing the pandemic in Ontario and Quebec.

Although out of scope of this paper, it is important to acknowledge the federal government played a crucial role in funding COVID-19 scientific research through the Canadian Institutes for Health Research. Of vital importance has also been the federal government leveraging its unique spending power to finance the bulk of the PT public health measures and economic recovery. Specifically, the federal government provided major safety net supports for individuals and businesses throughout the pandemic, and financed over 90% of the total costs of the pandemic response (Macdonald, 2021). The most wide-reaching of the economic supports for Canadians was the Canada Emergency Response Benefit (CERB), which provided CA$2000 monthly to individuals whose income was impacted by COVID-19 from March to September 2020 (Parliamentary Budget Officer, 2020). In the future, the federal government could leverage its spending power to provide even greater financial supports to provinces, such as to increase federal transfers, or even to help alleviate provincial debt or in light of projected declines in provincial revenues of roughly 10% (Béland *et al*., 2020a).

It is interesting to note that the federal government has, as of April 2021, not declared a national ‘public welfare emergency’ under the *Emergencies Act* (Canada, 2003). Such a declaration would increase the federal government's jurisdiction significantly, including powers to control supply and distribution of essential goods and services, and establish emergency shelters and hospitals. Some experts argued early in the pandemic that the declaration of a national state of emergency would have addressed some of the challenges with their capacity to test, trace, treat and isolate that were experienced in the PTs (Flood and Thomas, 2020). Due to the political legacy of this Act in terms of how it was used by the federal government against a radical separatist group in Quebec in 1970, the federal government has been reluctant to invoke this Act. That said, the PT governments quickly used their own emergency legislation to declare states of emergency and to impose a range of restrictions. Moreover, the federal government was able to use other existing legislation, such as the *Quarantine Act* to enforce international border measures. The political risks of declaring a national emergency thus outweigh the potential public health benefits. As noted by Fierlbeck and Hardcastle (2020: 46–47), ‘any intemperate exercise of federal emergency powers would be seen as intrusive, pernicious, illegitimate, and fundamentally destructive of intergovernmental relations in Canada’.

One year into the pandemic, the country is still struggling to contain the growth in viral transmission while, at the same time, manage the distribution of vaccines. Vaccine regulation is under federal jurisdiction (Health Canada) and the federal government has taken the lead on procurement and distribution (to the PTs) for COVID-19 vaccines, with heavy reliance on strong FPT coordination to ensure rapid roll out across the country. Despite securing more doses of multiple vaccines than any other country per capita (enough to immunize 335% of the Canadian population, as of 9 March 2021) and the first Canadians being vaccinated (outside of a clinical trial) in December 2020, roll-out of PT vaccination programs were slow between January and March 2020 (Coletta, 2020; Bloomberg, 2021). Yet, as of 27 April 2021, an estimated 30% of Canadians had received at least one vaccine dose in comparison with 42% in the United States and 50% in the UK (Our World in Data, 2021). Although the National Advisory Committee on Immunization (NACI) released recommendations for the prioritization of groups (NACI *et al*., 2020), the prioritization and distribution of COVID-19 vaccines varied widely across the country. Importantly, the lack of a national vaccine registry presents another major hurdle for Canadian decision-makers and researchers.

Although the early responses to the pandemic, supported by regular FPT strategic and technical committee discussions, were relatively well coordinated across the country, there were challenges in implementing effective border controls, ensuring coordinated communications strategies, addressing regional variations in capacity for testing, tracing, isolation and supports, and managing the crisis in LTC. Although these challenges cannot be addressed by the federal government alone, arguably a strengthened federal role is needed in the context of a persistent and major public health threat that appears to have pushed the limits of PT governments' capacity to respond.

Perhaps the biggest failing involved information sharing and the inability to maintain an adequate public health surveillance system to support local and PT decision making and effective contact tracing and case management systems. PHAC relies on the voluntary sharing of crucial PT COVID-19 case, death and health care utilization data to enable national surveillance and epidemiological modeling. The variations in the timing and content of data shared with the federal government highlighted the inadequacies with the current system, which have been well known for decades (Attaran and Houston, 2020). In the absence of a national data system, much of the data underpinning national surveillance was curated by self-organized, volunteer groups.^12^ Furthermore, a recent internal audit of PHAC's pandemic surveillance system detailed how Canada's epidemiological intelligence data network (the Global Public Health Intelligence Network), once praised by the World Health Organization (WHO) and supported one-fifth of the WHO epidemiological intelligence, met a similar fate similar to the rest of Canada's data infrastructure, having been severely scaled back as a result of budget cuts and restructuring in the years leading up to the pandemic (Robertson, 2020; PHAC, 2020g; Auditor General of Canada, 2021). Most acutely relevant to effective public health practice during the COVID-19 pandemic has been the high percentage of cases without any confirmed epidemiological link, thereby limiting the ability of public health officials to ascertain the highest risk events, spaces and activities – and intervene accordingly. In their recent review of Canada's pandemic response, the CPHA recommended a national framework for allocating public health human resources (e.g. for surges in contact tracing), principles for electronic contact tracing and standardized data collection tools (CPHA, 2021). The benefits of a strengthened national data infrastructure would be multifold: it would permit more accurate and timely data sharing with international partners, such as the WHO, to enable effective global surveillance; and it could enhance PT governments in their own epidemiologic modeling to inform local policy decisions. Drawing on national data, such modeling could help account for the cross-country mobility of people (and corresponding virus transmission) and could enable rapid sharing across PTs of spatiotemporal changes in viral transmission and possible trajectories, such as with the emergence of several variants of concern in early 2021.

Many of the challenges faced in Canada during the first year of the COVID-19 pandemic are not new to decision-makers and researchers. These challenges were identified in the 2003 Commission on SARS, including deficiencies in surge capacity; timely scaling of and access to laboratory testing and results; data access protocols and data sharing among levels of government; capacity for outbreak investigations; coordination across institutions; training, auditing and implementation in infection prevention and control protocols; and national electronic surveillance systems, as well as weak links between medical and public health systems and uncertainties around data ownership (Health Canada, 2003). In mid-April 2020, it was announced that two Senate committees had already begun to examine issues related to the COVID-19 pandemic and a third would be tasked with ‘Lessons Learned from the COVID-19 Pandemic and Future Preparedness’ (Senate, 2020). If Canada does not act on these lessons, they may fall to the wayside in a manner similar to response to lessons identified in the 2003 SARS inquiry.

Moreover, this pandemic has revealed fundamental weaknesses in the financing, regulation and management of LTC facilities across Canada, a sector that the federal government could, for the first time, play some role in the near future. Experts have expressed the urgent need for action to address these in the short term, but more fundamental, longer-term changes are required to address issues with its workforce, physical infrastructure and adherence to quality and safety standards (Ontario Auditor General, 2020; Marchildon and Tuohy, 2021). There is a well-accepted role for the federal government in supporting the financing of PT efforts to reform their LTC systems, its role could be even stronger in establishing quality standards and holding PT governments accountable for meeting these standards that may be warranted given the crisis in LTC that the COVID-19 pandemic has exposed.

In the future, FPT governments and their respective public health agencies may create more effective vehicles for pan-Canadian coordination and the sharing of information as well as mechanisms to deal with any potential policy conflicts concerning access to needed medical equipment and interprovincial transportation of people, goods and services. These efforts could build on the evidence of collaboration throughout the pandemic to date (Canada, 2020d). For example, Alberta was quick to share their surplus personal protective and medical equipment when Ontario and Quebec faced potential shortages during the first wave (Ontario, 2020b). A key challenge going forward will be finding the right balance between coordinated and cohesive approaches, while maintaining adequate regional flexibility in response to the varied impact of any subsequent pandemic waves in 2021. Some have suggested that a national approach to public health, e.g. through national framework(s) or legislation with clear FPT roles and responsibilities, could alleviate many of these concerns, while still respecting PT responsibilities (CPHA, 2021).

More broadly, governments and public health experts have learned there is need for enhanced public communication, transparency and trust. The pandemic has also highlighted the risks of mis- and disinformation, and the impacts this can have on effective public health interventions. Clear and transparent communication is paramount to ensuring public support of, and compliance with, restrictive public health measures and to address vaccine hesitancy. The federal government is uniquely positioned to leverage its leadership role in communicating with the Canadian public, providing a unifying vision, and coordinating messages with the other PT leaders. Yet, the vision set out by the federal government has been insufficient to unite the public, and contrasts with exemplar jurisdictions like New Zealand, Australia and South Korea which recognized the power of setting ambitious targets of virus elimination early in the pandemic – in contrast to the Canadian vision of merely planking the curve. In addition to managing future pandemics, failing to act upon these lessons will prove detrimental to the effective responses to other emerging public health, national and global emergencies, such as the climate crisis. Unlike Canada's experiences with SARS, the hope is that the lessons learned and any recommendations following the COVID-19 pandemic, including for a strengthened role for the federal government, will be acted upon swiftly before the policy window of opportunity for major reform is closed.

## Footnotes

1 In this paper, we focus mainly on the role of the federal government, although we provide some provincial data from the three most populous provinces of Ontario, Quebec and British Columbia (BC), whose combined populations constitute about 75% of the Canadian population (37.7 million, 2020) (Statistics Canada, 2021). These provinces are not meant to represent the Canadian experience given significant regional variations.

2 As of 9 March 2021, 293,860 (33%) of Canada's 893,518 cases and 10,493 (47%) of the 22,304 COVID-related deaths had been in Quebec; 311,112 (35%) cases and 7,083 (32%) deaths had been recorded in Ontario (CCODWG, 2021).

3 One of these provinces, Newfoundland and Labrador, implemented strict travel bans and their leaders and populace had a low tolerance for implementing additional public health measures (NAO, 2020). It is no coincidence the province had an impressive 43-day COVID-free streak during the summer of 2020 (CCODWG, 2021).

4 For example, the highest number of new daily cases reported in Ontario during the first wave was 634 (23 April 2021). The latest record, set on 29 April 2021, was 5053 new cases. Alberta, a province representing 11.6% of the Canadian population set its latest single-day record of 2,433 on 1 May 2021 (CCODWG, 2021); notably, 21.9% of all Canadian cases recorded in November 2020 occurred in Alberta.

5 In general, there was scarce data to accurately investigate the disproportionate impact of the virus and inform targeted policies. One exception is Toronto Public Health which collected detailed demographic data on all of its cases, including race, income and household size since May 2020. Between 20 May and 16 July 2020, 83% of COVID-19 cases in Toronto occurred among racialized communities (PHAC, 2020f). A recent report from Canada's national statistical office, Statistics Canada, shows similar disparities in the burden of COVID-19-related mortality, with the greatest racial differences being observed in Toronto's (Ontario) South Asian and Montreal's (Quebec) Black communities (Statistics Canada, 2020).

6 For example, the country's three territories implemented strict travel bans and entry requirements, e.g. Nunavut enforces a mandatory, supervised quarantine prior to entry. Cases in the territories were extremely rare during the first wave and no deaths recorded until October 30 (CCODWG, 2021); however, Nunavut experienced a large outbreak spanning November and December 2020, after identifying its first confirmed case on 6 November 2020.

7 Exemptions were made for flights from the USA, the Caribbean, Central and South America and Mexico, as well as chartered, private and business flights (Transport Canada, 2020). However, in response to limited demand for these flights, the majority of flights from these locations landed in these four locations – despite being permitted to land elsewhere (Manitoba, 2021). A February 2021 order extended this order to include flights from these locations, with the only exemptions being made for flights from Saint Pierre and Miquelon and cargo-only flights (ibid).

8 Although the first point of care SARS-CoV-2 diagnostic test was approved by Health Canada in March 2020, they were limited in supply and distribution focused on geographically remote regions. Two other rapid tests were approved on 30 September and 5 October 2020, for a total of six Health Canada approved tests, allowing rapid tests to be distributed more broadly across the country; however, their use mostly targeting high-risk environments (e.g. LTC facilities) and hotspot regions (Leavitt and Gallant, 2020). For example, in early December 2020, BC began piloting the use of rapid diagnostic tests among residents of LTC facilities and health care workers, while Quebec deployed them to its hardest hit communities and international airport (Hunter, 2020; Laframboise, 2020). On 24 November 2020, Ontario announced it had already deployed roughly 98,000 rapid diagnostic tests to several hospitals and assessment centers in its remote and rural communities, with plans to send another 1.2 million to LTC facilities and high-risk workplaces (Ontario, 2020e). Rapid tests were deployed to other high-risk areas and populations in each PT; for example, Manitoba began rapid testing of teachers in January 2021 (Bernhardt, 2020). By 29 November 2020, the federal government had procured 38 million rapid tests, and had shipped approximately 5.5 million to the PTs; while some PTs, such as those mentioned above, were quick to deploy them, others had concerns regarding their accuracy and were hesitant to use them (Leavitt and Gallant, 2020).

9 Including the failed CA$147M ‘Panorama’ system (BC Auditor General, 2015). The Canada Digital Service was created to oversee the development of a mobile exposure notification application, COVID Alert (Scassa *et al*., 2020). However, uptake was limited, with two of the country's larger provinces (BC and Alberta) not having yet joined as of April 2021 (Canada, 2020c). Further complicating matters, Alberta had previously launched its own app; however, it had reportedly traced only 20 cases over its first 6 months (Fletcher, 2020). Similarly, as of 22 November 2020, reportedly only 3406 Ontarians (i.e. roughly the equivalent of 2.5 days of new COVID-19 cases in November) had used the COVID Alert app to notify contacts of potential exposure (Ontario, 2020a, 2020b). Despite over 88% of Canadian adults having a smartphone, access was lower among higher-risk Canadians, such as those over 65 years of age (60%) (Scassa *et al*., 2020).

10 During wave one (1 March– 1 August 2020), the number of COVID-19-related deaths among LTC and retirement home deaths residents was 58 per 100,000 population in Quebec, 14 in Ontario and three in BC. During wave two (1 September 2020–15 February 2021), mortality declined in Quebec (33 per 100,000) but increased in Ontario and BC (16 and 12 per 100,000, respectively). Nationally, 20 per 100,000 LTC and retirement home residents died with COVID-19 during waves one and two (CIHI, 2021).

11 Cohorting refers to the grouping of residents and staff based on risk of infection and preferably isolating infected patients in a single occupancy room or designated area of the facility, when single occupancy rooms are limited.

12 Examples include the COVID-19 Canada Open Data Working Group (CCODWG), founded by two PhD epidemiology students of the University of Toronto, and the National Institute on Aging (NIA) Long-Term Care COVID-19 Tracker, curated by a think tank at Ryerson University in Toronto (CCODWG, 2021; NIA, 2020).

## Supplementary material

For supplementary material accompanying this paper visit http://dx.doi.org/10.1017/S1744133121000220. click here to view supplementary material
