# Supplementary material for: The federal government and Canada's COVID-19 responses: from ‘we're ready, we're prepared’ to ‘fires are burning’
Source: Health Econ Policy Law. 2021 Jun 22:1–19. doi: 10.1017/S1744133121000220 (PMC8326669; doi:10.1017/S1744133121000220)
Supplement: Supplementary file 1 [file S1744133121000220sup001.docx]

**Supplementary Materials: Tables 1-3**

**Table 1.** Overview of relevant demographics, and health and COVID-19 response institutions in Canada, BC, Ontario, and Quebec

| **Characteristic** | **Canada** | **British Columbia** | **Ontario** | **Quebec** |
| --- | --- | --- | --- | --- |
| **DEMOGRAPHICS (2020)** | | | | |
| Population | 37.6 M | 5.1 M | 14.7 M | 8.6 M |
| Population density | 4.0 per km^2^ | 5.0 per km^2^ | 14.8 per km^2^ | 6.0 per km^2^ |
| Pop. 65+ years old | 18.2 per cent | 15.7 per cent | 17.6 per cent | 18.3 per cent |
| **RELEVANT PUBLIC HEALTH AND HEALTH CARE INSTITUTIONS** | | | | |
| Public health institutions | The Minister of Health oversees a health portfolio comprised of Health Canada, the Public Health Agency of Canada (PHAC) and three other health-related institutions.  PHAC, established in 2004 following the SARS epidemic. One of its mandates is to respond to public health emergencies, including infectious diseases. | Overseen by the Provincial Health Officer and Ministry of Health (MOH). Shared responsibility between the Provincial Health Services Authority (PHSA), five Regional Health Authorities (RHAs) and First Nations Health Authority.  The BC Centre for Disease Control (BCCDC) manages public health programs and the BCCDC Public Health Laboratory. | Overseen by the MOH and Chief Medical Officer of Health (CMOH).  Public Health Ontario (PHO), established in 2008 following Toronto’s 2003 SARS outbreak, is responsible for coordinating the efforts of 34 public health units (PHUs) and the provincial laboratory services (PHO Laboratories, PHOL). | Overseen by the National Public Health Director/ Assistant Deputy Minister of Public Health. The 18 RHAs are overseen by the Ministry of Health and Social Services, Ministère de la Santé et des Services Sociaux (MSSS).  The National Institute of Public Health, Institut Nationald de Santé Publique du Québec (INSPQ), centralizes several public health activities, including laboratory services. |
| **PANDEMIC RESPONSE INSTITUTIONS** | | | | |
| Existing pandemic response plan, date | Canadian Pandemic Influenza Preparedness: Planning Guidance for the Health Sector, August 2018 | BC’s Pandemic Influenza Plan, February 2020. | Ontario Health Plan for an Influenza Pandemic, March 2013. | The Québec Pandemic Influenza Plan-Health Mission, March 2006. |
| COVID-19 Leadership | Unknown, other than the CPHO and Prime Minister | Provincial COVID-19 Task Force | COVID-19 Command Table | Unknown, other than Premier Legault |
| Other task force(s) | Special Advisory Committee (SAC); Technical Advisory Committee (TAC); and the COVID-19 Immunity Task Force | Yes, a provincial economic task force exists (Economic Recovery Task Force), the Provincial Emergency Management Office, and multiple topic- and geography-specific task forces. | Yes, a “Collaboration Table” exists, with representatives from multiple ministries, as well as specific sub-tables and topic- and geography-specific task forces. | No evidence of any formal provincial command tables or task forces evident in the public sphere. |

Sources: North American COVID-19 Policy Response Monitor. Available online: <https://ihpme.utoronto.ca/research/research-centres-initiatives/nao/covid19/> Accessed: Nov 20, 2020; Statistics Canada (2020). Population and demography statistics. Available online: [www.statcan.gc.ca/eng/subjects-start/population_and_demography](http://www.statcan.gc.ca/eng/subjects-start/population_and_demography) Accessed: Nov 20, 2020

**Table 2.** Overview of relevant public health measures taken in Canada, BC, Ontario, and Quebec

| **Characteristic** | **Canada** | **British Columbia** | | **Ontario** | **Quebec** |
| --- | --- | --- | --- | --- | --- |
| Date first confirmed case identified | January 25, 2020 | January 28, 2020 | | January 25, 2020 | February 28, 2020 |
| State of emergency | Not yet declared | First declared: March 17 | First declared: March 17 | | First declared: March 13 |
| Initial bans on public gatherings | March 16: PHAC recommends gatherings >50 people be cancelled. | March 12: <250 people  March 16: <50 people | March 13: <250 people  March 16: <50 people  March 28: <5 people | | March 13: <250 people  March 22: all indoor and outdoor gatherings |
| Domestic travel recommendations | No domestic travel bans implemented federally; however, international border restrictions, recommendations and precautions first introduced on January 25, 2020  April 20: Minister of Transport requires all air travelers to wear a non-medical face mask and mandatory pre-boarding health checks, including temperature screening  June 3: above order expanded to include trains, ships and transit. | March 2020: Only essential travel recommended, especially for rural communities  Mid-March through Mid-April 2020: Alberta, Yukon and Northwest Territories borders closed to non-essential travel  Mid-March through Mid-April 2020: borders closed to several remote Indigenous communities | No official policies  Late March through May: Residents asks to avoid non-essential travel to cottages (some regions took more active approaches to deter recreational travel, such as turning off utilities to seasonal properties) | | March 29: provincial border restrictions and checkpoints enforced, all residents asked to avoid non-essential inter-regional and -city travel  April 1: ban on non-essential travel into certain remote and/or Indigenous regions  April 4: ban on non-essential travel into certain areas of Northern Quebec, enforced with policed checkpoints (e.g., 14 CAF patrols deployed to Nunavik) |
| Travel advice given to the public prior  to March break | Travelers should check with advisories and “think carefully” about their travel plans. Since February 9, travel on cruise ships has been discouraged. Since March 13, the federal government has recommended avoiding all non-essential travel, especially to high-risk areas. | CMOH warns of travel risks and discourages “all non-essential travel.” Anyone who travels abroad must stay home from work or school for 14 days upon their return to the province. | Premier tells residents to “go away and have a good time”. No travel-related quarantine measures recommended by CMOH until March 16. | | Health authorities advised travelers to self-monitor and, if symptomatic, contact health authorities. Only travelers from Hubei, China and Iran were asked to quarantine; travelers from other locations asked to self-monitor. |
| School closures | No guidance provided | March 17; i.e., during March break | March 23; i.e., immediately after March break | | March 13; i.e., one week after March break |
| Definition of essential businesses | No guidance provided | “Daily services essential to preserving life, health, public safety and basic societal functioning.”  A detailed list was provided by provincial authorities. | No specific definition, but the province provided a detailed list including supply chains, retail, accommodations, food services, transportation, communications, etc. | | “Work environments providing the priority services” and supporting the “the minimum activity needed to ensure the future resumption of the activities of enterprises providing non-priority services, excluding commercial enterprises.” A detailed list was provided . |
| Closure of non-essential businesses | No guidance provided | March 17: non-essential closed  March 20/21: dine-in eating and personal care services closed  March 26: detailed list of essential businesses released | March 23: initial list (74 items)  April 3: stricter list (44 items) | | March 15: all bars and restaurants ordered to restrict done-in services to 50 per cent capacity (or take-out only)  March 23: only “priority” services permitted |
| Face mask recommendations | May 20: Following Prime Minister’s comments on his intentions to wear a face masks, CPHO Dr. Tam first officially recommends the use of non-medical face masks when maintaining a physical distance is not possible. | CMOH “expects” everybody able to wear a mask to do so in crowded spaces, especially where distancing cannot be maintained, but states that no mandatory mask policies will be introduced (comments made up until Nov 17, 2020)^2^  Nov 19: Mandatory face mask policy for all public indoor and retail spaces (for persons aged 2+)^2^ | June-July: most PHUs mandate mandatory mask policies; provincial authorities state that province-wide policy is “unnecessary”  Oct 2: Province mandates mandatory face mask policy for all indoor public spaces (for persons aged 2+) | | July 1: A Montreal suburb, Côte Saint-Luc, enacts the province’s first mandatory face mask policy  July 18: mandatory mask policy enacted, for all enclosed or partially enclosed public places throughout the province (for persons aged 12+). |
| Recommendations for travel-related quarantine (14 days) | Feb 3^4^: Quarantine required under the *Quarantine Act* for all repatriated persons from Hubei, China; returning travelers were housed at a military base near Toronto  Feb 8: All travelers returning from Hubei (voluntary)  Mar 25: all international travelers required to quarantine, with the exception of essential workers  Apr 14: Emergency order stipulating that persons in quarantine cannot quarantine in place with vulnerable persons  Nov 21: All inbound international travelers are required to submit a digital quarantine plan prior to arrival through the ArriveCAN application, subject to fines up to $1,000; travelers must also confirm arrival at their final place of quarantine within 48 hours of entering Canada  Feb 2021 (TBD): Mandatory 3-day quarantine in a designated government hotel, at the traveler’s expense, for international air travelers  Federal guidance uses “quarantine” and “self-isolation” interchangeably | Feb 4-6: Travelers, and close contacts, from Hubei (voluntary)  Feb 24: all international travelers asked to self-monitor for 14 days  Mar 2: all travelers from China and Iran (voluntary)  Mar 12: all travelers (voluntary)  Apr 8: All travelers entering the province legally required to submit a quarantine plan; individuals without an adequate plan taken to a quarantine location until adequate plans provided. | March 16: CMOH advises isolation for immune-compromised persons and those aged 70+ years.   - March 16-19: Quarantine recommended for arriving travelers. | | Feb 6: Individuals arriving from Hubei, China (voluntary)  Mar 8: all travelers asked to self-monitor; travelers from Hubei and Iran to quarantine (voluntary)  Mar 12: all symptomatic travelers (voluntary); mandatory for all public service staff and for all individuals offering health care, education, and child care services.  Mar 25: all travelers required to quarantine |
| Travel-related testing requirements | Jan 7, 2021: Mandatory proof of negative PCR test within 72 hours of boarding for all international air travelers aged 5+ years  Feb 15, 2021: Mandatory proof of negative PCR test within 72 hours of boarding for all inbound travelers crossing the US-Canada land border  Feb 22, 2021: Mandatory test required upon arrival at 1 of 4 designated airports, at the traveler’s expense; travelers must wait for their test result at a designated quarantine hotel (up to 3 days). Persons found positive for SARS-CoV-2 must spend the remainder of their 14-day quarantine in a designated government isolation center; otherwise, they can return to their normal place of quarantine. Land border travelers are exempt from the mandatory hotel quarantine but must take a diagnostic test on arrival. All travelers are required to be tested again at the end of the 14-day quarantine period. | Jan 6, 2021: pilot of a free and voluntary testing program launched at Toronto’s international airport for inbound international travelers  Feb 1, 2021: Mandatory test required upon arrival at Toronto’s international airport (i.e., the only airport in Ontario permitted to accept international flights) for all travelers aged 5+ years whose final destination is Ontario | None | | None |

Sources: North American Observatory on Health Systems and Policies (NAO) (2020). North American COVID-19 Policy Response Monitor. Available online: <https://ihpme.utoronto.ca/research/research-centres-initiatives/nao/covid19/> Accessed: Nov 20, 2020; Government of BC (2020). Daily COVID-19 update; Government of BC (2020). Province-wide restrictions. Available online: <https://www2.gov.bc.ca/gov/content/safety/emergency-preparedness-response-recovery/covid-19-provincial-support/restrictions> Accessed: Nov 20, 2020; Timeline of Canadian Border Services Agency’s COVID-19 border measures: Public Safety Canada (2020). Timeline – CBSA Border Measures. Available online: <https://www.publicsafety.gc.ca/cnt/trnsprnc/brfng-mtrls/prlmntry-bndrs/20201119/015/index-en.aspx> Accessed: Feb 8, 2021

**Table 3.**Measures taken to mitigate the impact of COVID-19 in Long-term Care (LTC) facilities in Canada, BC, Ontario, and Quebec

| **Characteristic** | **Canada** | **British Columbia** | **Ontario** | **Quebec** |
| --- | --- | --- | --- | --- |
| **ORGANIZATION** | | | | |
| Authority | Under PT authority but Health Canada provides guidance, and federal government may provide support by invitation | MOH | Ministry of Long-Term Care (MLTC) | MSSS |
| Public/Private mix  (2020) | 28 per cent private, 23 per cent non-profit, 46 per cent publicly owned | 34 per cent private, 28 per cent non-profit, 38 per cent publicly owned | 57 per cent private, 27 per cent non-profit, 16 per cent publicly owned | 14 per cent private, 86 per cent publicly owned. Breakdown of private for profit and not-for-profit not currently available. |
| **COVID-19 IMPACT IN LTC FACILITIES, AS OF NOVEMBER 24** | | | | |
| Per cent of total cases | 12 per cent | 4 per cent | 13 per cent | 16 per cent |
| Per cent of total deaths | 75 per cent | 44 per cent | 70 per cent | 81 per cent |
| LTC homes affected | 31 per cent | 30 per cent | 45 per cent | 36 per cent |
| Military deployment | April 17: Deployment of Canadian Armed Forces (CAF) personnel and resources to LTC facilities in need of support.  June 26: Announcement CAF would be recalled, and the Canadian Red Cross would be deployed to assist homes in further need of support | No support from CAF requested thus far. | April 22: CAF deployed to 5 LTC facilities  May 26: CAF deployed to 7 LTC facilities  June 2 and 4: Mandatory management orders appointing 2 hospitals to manage 2 LTC facilities  July 3: Final CAF team leaves  October 10: Red Cross deployed to 7+ homes | April 20: CAF personnel deployed  April 22: An additional 1,000 CAF personnel deployed across 47 affected facilities  July 6: CAF leaves, Red Cross deploys 900+ personnel to provide support until homes are designated “green”, according to Quebec’s classification system. |
| **KEY COVID-19 RESPONSE MEASURES, AS RECOMMENDED BY PHAC** | | | | |
| Visitation restrictions | April 17: Federal guidance recommends limiting to essential visits only | March 11: Advised extra caution to be taken  March 17: Restricted to essential visits | March 11: Screening required for visitors and only essential visits recommended  March 23: Residents prohibited from leaving the facility for short visits | March 8: Travelers asked to not visit for 14 days  March 14: Limited to essential  March 23: “Lockdown” prohibiting residents from having any visitors or outside access |
| Zoning for COVID-19 patients | Cohorting recommended, with exclusive staffing, particularly where single rooms are not available. | Confirmed/suspected cases are isolated in private room. | Confirmed/suspected cases are isolated in private or cohorted room. | Buffer zones created within existing facilities. Symptomatic and positive residents placed in designated “hot zones”. |
| One staff to one facility policies | April 13: Measures recommended to promote staff to limit their work to one facility, e.g., increasing full-time positions.^4^ | Introduced on March 27.  Two RHAs implemented policies earlier: March 21/22. | Introduced on April 14. | April 10: employees assigned to hot zones must limit their movement between units.  October 28: General directive issued requiring staff to limit work to one facility. |
| Screening of staff and residents | Testing and screening of all staff and residents recommended for facilities experiencing an outbreak. | March 21-27: Active screening of staff recommended  April 5: symptomatic staff of LTC included in testing eligibility | March 30: recommended screening and temperature checks for all staff  April 10: Prioritized testing of symptomatic frontline healthcare staff  April 15: the above expanded to include asymptomatic LTC staff | April 2: Screening prioritizes symptomatic health staff in direct contact with patients.  April 8: “Systematic” testing of all LTC residents and staff  May 4: MOH spokesperson stated asymptomatic LTC staff can be tested (however, conflicting reports have been published by media sources) |

 Sources: North American Observatory on Health Systems and Policies (2020). North American COVID-19 Policy Response Monitor. Available online: <https://ihpme.utoronto.ca/research/research-centres-initiatives/nao/covid19/> Accessed: Nov 20, 2020; Canadian Institute for Health Information (2020). Long-term care homes in Canada: How many and who owns them? Available online: [www.cihi.ca/en/long-term-care-homes-in-canada-how-many-and-who-owns-them](http://www.cihi.ca/en/long-term-care-homes-in-canada-how-many-and-who-owns-them) Accessed: Nov 20, 2020;
National Institute on Ageing (2020). LTC COVID-19 Tracker. Available online: <https://ltc-covid19-tracker.ca/> Accessed: Nov 20, 2020; PHAC (2020). Public Health Agency of Canada releases interim guidance for infection prevention and control of COVID-19 for long-term care homes. Available online: [www.canada.ca/en/public-health/news/2020/04/public-health-agency-of-canada-releases-interim-guidance-for-infection-prevention-and-control-of-covid-19-for-long-term-care-homes.html](http://www.canada.ca/en/public-health/news/2020/04/public-health-agency-of-canada-releases-interim-guidance-for-infection-prevention-and-control-of-covid-19-for-long-term-care-homes.html). Accessed: Feb 7, 2021
